# Association of Barley Root Elongation with ABA-Dependent Transport of Cytokinins from Roots and Shoots under Supra-Optimal Concentrations of Nitrates and Phosphates

**DOI:** 10.3390/cells10113110

**Published:** 2021-11-10

**Authors:** Lidiya Vysotskaya, Leylya Timergalina, Guzel Akhiyarova, Alla Korobova, Vadim Fedyaev, Igor Ivanov, Guzel Kudoyarova, Dmitry Veselov

**Affiliations:** 1 Laboratory of Plant Physiology, Ufa Institute of Biology, Ufa Federal Research Centre of the Russian Academy of Sciences, Pr. Octyabrya, 69, 450054 Ufa, Russia; vysotskaya@anrb.ru (L.V.); l.n.timergalina@anrb.ru (L.T.); akhiyarova@rambler.ru (G.A.); muksin@mail.ru (A.K.); vadim.fedyaev@gmail.com (V.F.); i_ivanov@anrb.ru (I.I.); veselov@anrb.ru (D.V.); 2 Department of Biology, Bashkir State University, Zaki-Validi St. 32, 450074 Ufa, Russia

**Keywords:** *Hordeum vulgare* L., Az34 barley mutant, abscisic acid (ABA), cytokinins, mineral nutrition, root elongation

## Abstract

Changes in root elongation are important for the acquisition of mineral nutrients by plants. Plant hormones, cytokinins, and abscisic acid (ABA) and their interaction are important for the control of root elongation under changes in the availability of ions. However, their role in growth responses to supra-optimal concentrations of nitrates and phosphates has not been sufficiently studied and was addressed in the present research. Effects of supra-optimal concentrations of these ions on root elongation and distribution of cytokinins between roots and shoots were studied in ABA-deficient barley mutant Az34 and its parental variety, Steptoe. Cytokinin concentration in the cells of the growing root tips was analyzed with the help of an immunohistochemical technique. Increased concentrations of nitrates and phosphates led to the accumulation of ABA and cytokinins in the root tips, accompanied by a decline in shoot cytokinin content and inhibition of root elongation in Steptoe. Neither of the effects were detected in Az34, suggesting the importance of the ability of plants to accumulate ABA for the control of these responses. Since cytokinins are known to inhibit root elongation, the effect of supra-optimal concentration of nitrates and phosphates on root growth is likely to be due to the accumulation of cytokinins brought about by ABA-induced inhibition of cytokinin transport from roots to shoots.

## 1. Introduction

The sessile lifestyle of plants limited by the site of their germination is compensated by the ability of their roots to grow quickly, which is important for optimizing nutrient acquisition. Since nitrates are the predominant form of nitrogen (N) acquired by most crops, they are highly mobile and therefore frequently leach to lower layers of the soil. Under these conditions, efficient nitrate acquisition depends on root steep elongation, increasing rooting depth [1,2]. However, fast root elongation is not always beneficial and shallow roots enable acquisition of topsoil resources, which include immobile phosphates, as well as mobile resources that have not yet been depleted from the topsoil by leaching. In this case, slowing down the growth of roots deep into the soil becomes useful. Thus, adequate changes in root elongation are important for plant adaption to the changing root environment, and it is important to understand how plants sense concentration of ions in the soil and how the signals are transduced to changes in root elongation.

Availability of mineral nutrients influences hormone concentration in plants, while hormones regulate numerous processes, including plant growth and development. Therefore, hormones are believed to act as mediators in plant responses to the availability of nutrients. A specific role for cytokinin as part of nitrate signaling has been proposed [3,4]. It was shown that nitrate deficiency down-regulates expression of isopentenyl transferase (*IPT*) genes [5] responsible for cytokinin synthesis [6], while the addition of nitrates to the N-depleted medium results in up-regulation of these genes [7]. These responses depended on the function of nitrate sensors (NRT1), called transceptors, due to their dual function as nitrate transporters and receptors [8]. This conclusion was based on experiments with the mutants characterized by impaired NRT1 function and absence of either changes in expression of *IPT* genes [9] or root cytokinin content [10] under nitrate shortage.

Deficiency of nitrates and phosphates both influenced the expression of the *IPT* genes [11] and the content of cytokinins in plants [12], involving these hormones in the response of plants to P deficiency. The most attention has been paid to the transport of cytokinins from roots to shoots, acting as a long-distance signal that controls shoot function in response to the availability of nutrients (see the review of Kudoyarova et al. [13] and references therein). The involvement of hormones in the growth reaction of the roots attracted less attention. Nevertheless, the decline in root cytokinin of P- and N-starved plants was accompanied by acceleration of root growth [11,12,14]. Since cytokinins can inhibit root elongation [15], a decrease in their content was considered as the cause of accelerated root growth in response to deficiencies in mineral nutrients [16]. This suggestion was supported by the absence of root growth response in the *nrt1-1* mutant, in which nitrate starvation failed to change root cytokinin content [10].

Long-term effects of supra-optimal concentration of mineral nutrients on the hormone content and root growth have been less studied than the effects of nutrient starvation. One of few reports showed accumulation of cytokinins in a part of the roots exposed to high concentrations of mineral nutrients, imitating localized fertilizer placement, which was accompanied by inhibition of root elongation [14]. However, a mechanism of this effect remained unclear. 

Cytokinins and abscisic acid (ABA) are both involved in responses to the availability of mineral nutrients. Experiments with wheat plants treated with fluridone (inhibitor of ABA synthesis) and ABA-deficient barley plants showed that changes in cytokinin concentration in response to the shortage of mineral nutrients depended on the ability of plants to accumulate ABA [11,16]. However, interaction of ABA and cytokinins in response to long-term action of supra-optimal concentrations of mineral nutrients has not been studied. 

High nitrate concentrations increased ABA content in the root tips, which was associated with the observed changes in root growth [17]. Nevertheless, it is not easy to link ABA with the changes in root growth due to the discrepancy between the results obtained in experiments with exogenous ABA and experiments relating to root growth responses with the changes in the concentration of endogenous ABA. ABA treatment mostly inhibits root elongation [18], while, under some conditions, accumulation of endogenous ABA was accompanied by the acceleration of root growth [19,20]. This discrepancy can be explained if we take into account the interaction of ABA with other hormones. However, this has not been done frequently enough. Experiments with exogenous ABA do not allow strict control of the accumulation and distribution of this hormone in the plants. The use of ABA-deficient mutants is an attractive alternative that provides insight into the mechanisms of ABA action. This approach has been successfully used in the case of cytokinin deficient transgenic Arabidopsis. Phenotyping these plants confirmed the capacity of cytokinins to promote shoot growth and inhibit root growth of plants [15]. Previous experiments with ABA-deficient barley plants showed dependence of cytokinin metabolism and root growth on the capacity of phosphate-starved plants to accumulate ABA [11]. The goal of the present research was to test a hypothesis that ABA is involved in the control of root growth and cytokinin distribution between roots and shoots grown under supra-optimal concentration of mineral nutrients. This was achieved by the comparison of growth and cytokinin content in ABA-deficient barley plants and their parental cultivar exposed to high (300%) concentration of Hoagland-Arnon (H-A) solution. We decided to study increased concentration of all components of the H-A solution and not individual ions, so as not to disturb the balance between mineral nutrients, which could otherwise affect their uptake by plants [21].

## 2. Materials and Methods

### 2.1. Plant Growth Conditions 

Seeds of barley *Hordeum vulgare* L. (ABA-deficient mutant Az34 and its wild-type cv. Steptoe) were sterilized in a 2% NaClO solution for 10 min. The seeds were then washed with running tap water for 1 h. The seeds were laid out in a single layer on filter paper moistened with distilled water in a container, which was then covered with aluminum foil and placed for 1 day for seed stratification in a refrigerator at +4 °C. Afterwards the container stayed at room temperature for 1 day more, after which the seedlings were placed on sealed and tied together glass tubes, floated on tap water (200 seeds/2 L of water), and exposed to illumination of 340 µmol s^−1^ m^−2^ in a 14 h light period. At the age of 4 days, they were transferred to 10% and 300% Hoagland-Arnon nutrient medium. A 10% solution was chosen, since previous experiments showed that it was optimal for the growth of plants of this age [20]. At 2 days after the transfer to the Hoagland-Arnon salt solutions, 6-day-old plants were sampled for hormone analysis and transpiration. The growth was measured in 8-day-old plants.

### 2.2. Treatment with Cyanide M-Chlorophenylhydrazone (CCCP)

The importance of energizing cytokinin transport has been demonstrated with the help of carbonyl cyanide m-chlorophenylhydrazone (CCCP), a protonophore that collapses proton gradients across membranes, thereby inhibiting secondary active transmembrane transport [22]. CCCP was added to the nutrient solution of 6-day-old plants to yield 10 μM concentration. Roots and shoots were sampled for hormone analysis 1 h after the start of CCCP-treatment.

### 2.3. Xylem Sap Collection

To collect xylem sap, barley seedlings were cut under water and the shoots reconnected to the roots with fine silicon tubes [16]. Comparing the transpiration rate of intact, cut, and rejoined plants showed that this procedure did not significantly affect transpiration within 10 min. After 10 min of collecting root exudate, the collection tubes were disconnected and the root exudate within the tubes was weighed, frozen in liquid nitrogen, and stored at −80 °C prior to purification, separation on TLC, and determination of cytokinin concentrations with the help of immunoassay. Root export of cytokinins was calculated by multiplying the measured cytokinin concentration by the transpiration rate. 

### 2.4. Determination of the Transpiration Rate

Transpiration was determined by the weight method. To do this, plants were placed in plastic beakers (10 plants per beaker), covered with foil to prevent evaporation from the solution surface, and weighed every 15 min. According to the difference in the weight of the beaker, the amount of evaporated water was calculated as mg of water per plant per hour.

### 2.5. Hormone Extraction and Purification

Hormones were extracted from homogenized shoots and roots and collected xylem sap of barley plants with 80% ethanol overnight at 4 °C. Cytokinins and ABA were processed in different ways from aliquots of aqueous residue, as described by Vysotskaya et al. [11]. In short, CKs were concentrated on a C18 column (Waters, Milford, MA, USA), washed with water, and eluted with 5 mL of 80% ethanol. The dry residues dissolved in small amounts of ethanol were loaded on precoated 5 × 20 cm, 0.25 mm-thick silufol 60 F-254 plates (Merck, Darmstadt, Germany) for thin layer chromatography in the solvent system of butanol, ammonium hydroxide, and water (6:1:2) for separation of cytokinins bases and their derivatives. This procedure enabled separation and assay of cytokinin nucleotide (ZN, Rf 0–0.1), riboside of zeatin (ZR, Rf 0.4–0.5), and zeatin (Z, Rf 0.6–0.7) [23]. Eluates from the zones corresponding to the position of cytokinin standards were then immunoassayed. 

ABA was partitioned with diethyl ether from the aqueous residue after diluting with distilled water and acidification with HCl to pH 2.5. The hormones were then transferred from the organic phase into a solution of NaHCO_3_, re-extracted from the acidified aqueous phase with diethyl ether, methylated with diazomethane, and immunoassayed [20]. Reducing the amount of extractant at each stage and re-extraction increased hormone recovery’s selectivity [24].

### 2.6. Immunoassay

Immunoassay of zeatin, its riboside and nucleotide from plant tissues and xylem sap was carried out using an antiserum raised against zeatin riboside (ZR), shown to have high specificity to zeatin derivatives [23]. Cross-reactivity of anti-ZR serum to derivatives of other cytokinin bases (dihydrozeatin and isopentenyladenine) is low. This method has been proven to be reliable by testing its results against a physico-chemical assay [22]. 

For immunoassay of ABA, the corresponding antibodies were used. Antibody cross-reactivity to ABA, phaseic acid, and xanthoxin was 100%, 0.1%, and 0.001%, respectively.

Enzyme immunoassay was performed with the protocol, in which a conjugate of ZR or ABA to ovalbumin was absorbed onto the solid phase (wells of microplates). A mixture of standard or sample plus specific serum was added to each well and incubated. Unbound rabbit antibodies were washed away, and goat anti-rabbit IgG, conjugated to peroxidase, was incubated with the adsorbed antigen-antibody complex. All wells were washed again, and the substrate solution consisting of o-phenylene-diamine was added. Color developed was quantitated at 492 nm with a microphotometer.

### 2.7. Immunolocalization of Cytokinins in Root Tips

For immunolocalization, cytokinins were conjugated to proteins of the cytoplasm to prevent them from washing out during the dehydration process. Specifically, free cytokinin bases in tissues of root tips 5 mm long were fixed in a mixture 4% paraformaldehyde (Ridel-deHaen, Seelze, Germany) and 0.1% glutaraldehyde (Sigma, Steinheim, Germany), as described [22]. After washing with 0.1 M phosphate buffer (PB), tissues were dehydrated in a series of ethanol dilutions. After this, the tissues were embedded in methacrylate resin JB-4 (Electron Microscopy Sciences, Hatfield, PA, USA). Immunolocalization of hormones was carried out using antisera against ZR. Histological sections 1.5 μm thick were obtained using a rotary microtome HM 325, MICROM Laborgerate, Walldorf, Germany). After applying blocking solution (0.1 M PB containing 0.2% gelatin and 0.05% Tween-20) for 30 min, root sections were incubated with diluted rabbit anti-ZR sera (dilution 1:80) overnight at 4 °C. The slides were then washed three times in 0.1 M PB with 0.05% Tween-20, and anti-rabbit IgG secondary antibodies, conjugated to Alexa Fluor 555 (Invitrogen, Rockford, IL, USA), were applied for 3 h at 37 °C. The slices were then rinsed again five times with PB, covered, and then imaged by confocal microscopy with FV3000 Fluoview (FV31-HSD) (Olympus, Tokyo, Japan). The laser excitation line was 561 nm. Fluorescence emission was detected at 568 nm. Earlier, specificity and reliability of immunostaining was confirmed in experiments, where increased immunostaining was detected in the plants treated with exogenous hormones [22] or in transgenic plants with induced expression of the *ipt* gene, controlling cytokinin synthesis [25] (positive control). Non-immune rabbit serum was used as a control, and the absence of immunostaining when anti-ZR serum was substituted with the non-immune serum confirmed the reliability of the technique. The average fluorescence intensity of the images taken with the confocal microscope was estimated using the ImageJ software (National Institutes of Health, Bethesda, MD, USA, https://imagej.nih.gov/ij, accessed on 7 September 2021). The fluorescence was expressed in arbitrary units, with maximal staining fluorescence taken as 100% and minimal as 0%.

### 2.8. Statistical Analysis

The data were processed using the Statistica version 10 software (Statsoft, Moscow, Russia). In figures and tables, data are presented as mean ± standard error (SE). The significance of differences was assessed by ANOVA, followed by Duncan’s test (*p* < 0.05).

## 3. Results

**Figure 1:**
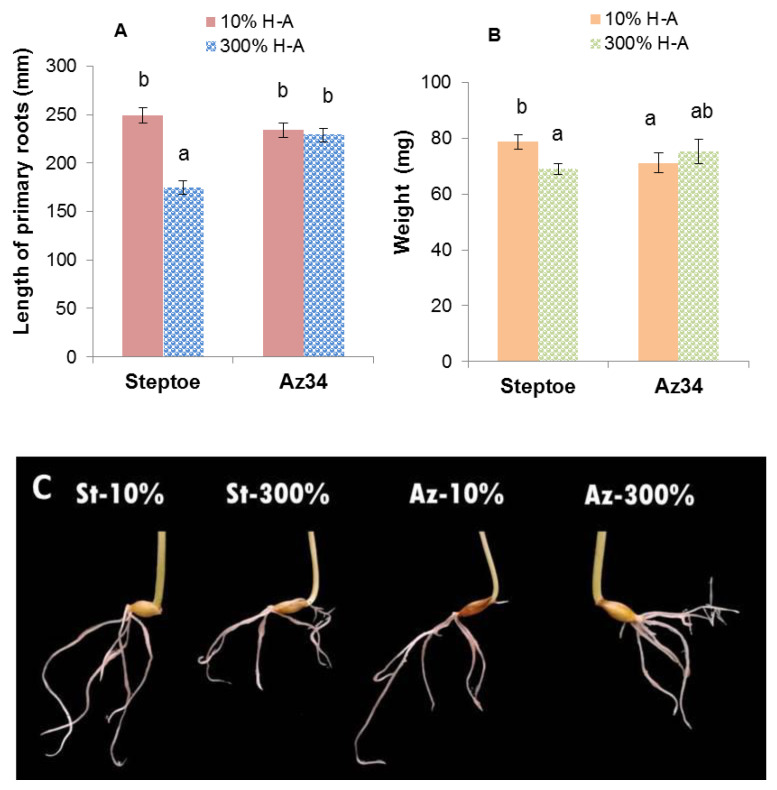
Total length of primary roots (**A**), weight (**B**), and root image (**C**) of 8-day-old barley wild type (Steptoe) and its ABA-deficient mutant (Az34) growing at 10% of supra-optimal (300%) Hoagland-Arnon solution (H-A). Means ± SE are presented. Statistically different means (n = 20) are indicated by different letters (*p* < 0.05, ANOVA, Duncan’s test).

Counting the number of main roots showed that it was similar in barley plants of both genotypes (about 7–9 per plant) and did not depend on the concentration of mineral nutrients in the solution. However, roots of Steptoe plants grown on supra-optimal 300% H-A solution were shorter and lighter than those of plants of the same genotype grown on 10% solution (Figure 1A,B). Meanwhile, there was no significant difference in the root length or weight of Az34 plants grown on solutions with different concentrations of mineral nutrients.

**Figure 2:**
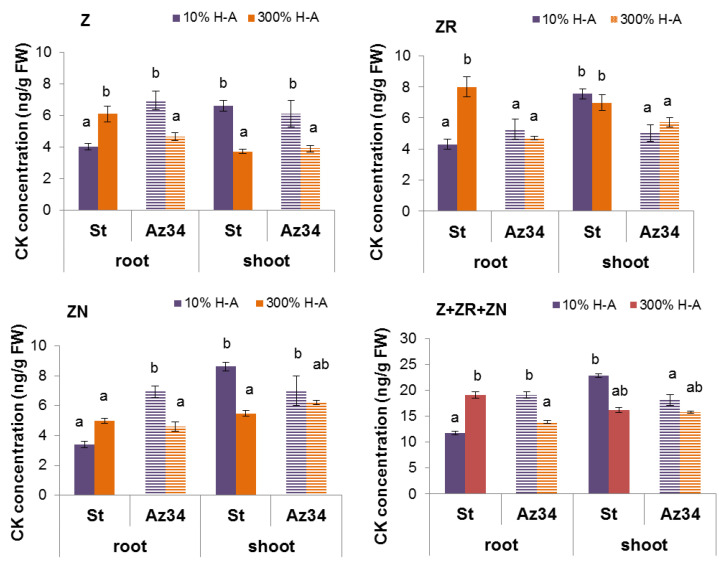
Concentration of zeatin (Z), zeatin riboside (ZR), zeatin nucleotide (ZN) and their sum (Z+ZR+ZN) in roots and shoots of 6-da-old barley wild type (Steptoe) and its ABA-deficient mutant (Az34) growing at 10% of supra-optimal (300%) Hoagland-Arnon solution (H-A). Means ± SE are presented. Statistically different means (n = 9) are indicated by different letters (*p* < 0.05, ANOVA, Duncan’s test).

**Figure 3:**
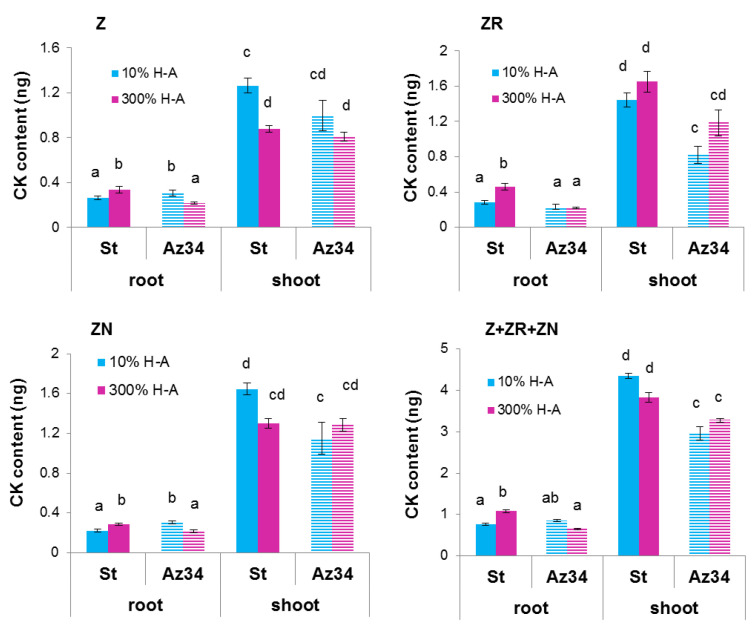
Content of zeatin (Z), zeatin riboside (ZR), zeatin nucleotide (ZN) and their sum (Z + ZR + ZN) in roots and shoots of 6-day-old barley wild type (Steptoe) and its ABA-deficient mutant (Az34) growing at 10% of supra-optimal (300%) Hoagland-Arnon solution (H-A). Means ± SE are presented. Statistically different means (n = 9) are indicated by different letters (*p* < 0.05, ANOVA, Duncan’s test).

Concentration of cytokinins (expressed as ng per g of fresh weight (FW), Figure 2) and its content in the whole organ (Figure 3) were higher in the roots of Steptoe plants fed with a high rather than low concentration of mineral nutrients. Concentrations of zeatin and its riboside were higher in the roots of Steptoe supplied with 300% H-A solution compared to 10% H-A solution with the greatest increase in the case of zeatin riboside. An inverse pattern was detected in shoots of Steptoe plants, where cytokinin concentration was higher at lower concentration of nutrients (Figure 2). Unlike Steptoe, the hormone level was neither increased in roots nor decreased in the shoots by the supra-optimal concentration of mineral nutrients supplied to Az34 plants. Concentrations of zeatin and its nucleotide in roots were even lowered by high nutrient concentration. 

Calculation of bulk root cytokinin content showed a similar pattern to that of cytokinin concentration (Figure 3): accumulation of cytokinins in the roots of Steptoe exposed to supra-optimal concentration of mineral nutrients, as well as the absence of the effect in Az34 plants.

The root-to-shoot ratio of total cytokinin content was increased about 1.5 times (from 0.18 to 0.28) by supra-optimal concentration of mineral nutrients in the case of Steptoe, while an inverse pattern was detected in Az34. 

**Figure 4:**
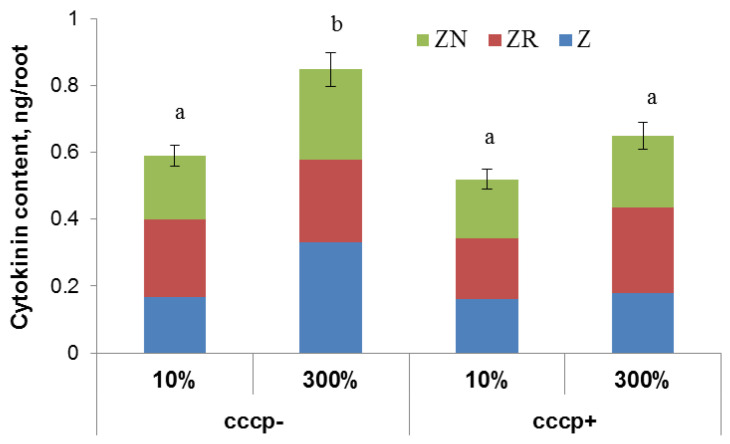
Total concentration of zeatin (Z), zeatin riboside (ZR), and zeatin nucleotide (ZN) in roots of 6-day-old barley wild type (Steptoe) growing at 10% of supra-optimal (300%) Hoagland-Arnon solution (H-A) 1 h after treatment with carbonyl cyanide m-chlorophenyl hydrazone (cccp). Means ± SE are presented. Statistically different means (n = 9) are indicated by different letters (*p* < 0.05, ANOVA, Duncan’s test).

Treatment of Steptoe plants with the protonophore carbonyl cyanide m-chlorophenylhydrazone (CCCP) prevented cytokinin accumulation in the roots of Steptoe plants grown at 300% H-A solution (Figure 4).

**Figure 5:**
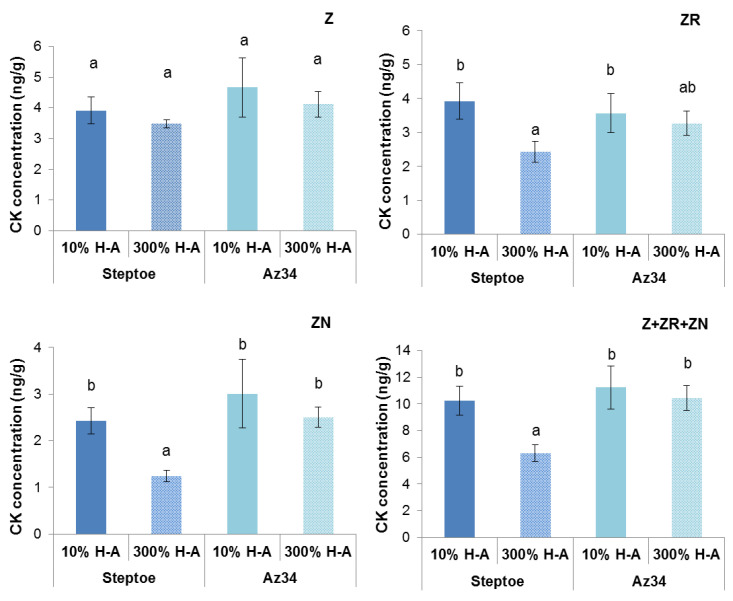
Concentration of zeatin (Z), zeatin riboside (ZR), zeatin nucleotide (ZN) and their sum (Z+ZR+ZN) in xylem exudate of 6-day-old barley wild type (Steptoe) and its ABA-deficient mutant (Az34) growing at 10% of supra-optimal (300%) Hoagland-Arnon solution (H-A). Means ± SE are presented. Statistically different means (n = 9) are indicated by different letters (*p* < 0.05, ANOVA, Duncan’s test).

Measurement of cytokinin concentration in the xylem sap showed a 1.6-fold lower level of zeatin riboside in the case of Steptoe plants grown on 300% H-A solution compared to 10% H-A solution (Figure 5).

Previous experiments showed that the procedure of xylem exudate sampling did not influence the rate of transpiration [16]. There was a 10% decrease in transpiration in Steptoe plants supplied with this supra-optimal concentration of nutrients (Table 1). As a result, calculation of cytokinin delivery by multiplying cytokinin concentration in the xylem sap by the transpiration rate showed even greater (1.8-fold) decline in the delivery of zeatin riboside from roots to shoots of Steptoe plants grown at 300% compared to 10% H-A solution. Neither the concentration of cytokinins in xylem sap nor the transpiration of Az34 barley mutant were altered by the level of nutrients, resulting in the unchanged level of cytokinin delivery from roots to shoots of the plants of this genotype.

**Figure 6:**
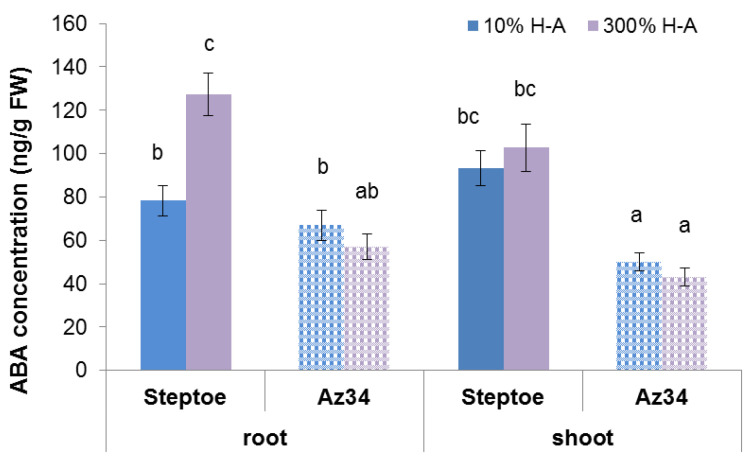
Concentration of ABA in roots and shoots of 6-day-old barley wild type (Steptoe) and its ABA-deficient mutant (Az34) growing at 10% of supra-optimal (300%) Hoagland-Arnon solution (H-A). Means ± SE are presented. Statistically different means (n = 9) are indicated by different letters (*p* < 0.05, ANOVA, Duncan’s test).

As expected, concentration of ABA was lower in ABA-deficient Az34 mutant s in its parent cultivar Steptoe. Root ABA concentration was increased in Steptoe plants grown on 300% H-A solution compared to 10% solution, while no significant difference in root ABA content was detected in Az34 plants grown at either nutrient level (Figure 6).

**Figure 7:**
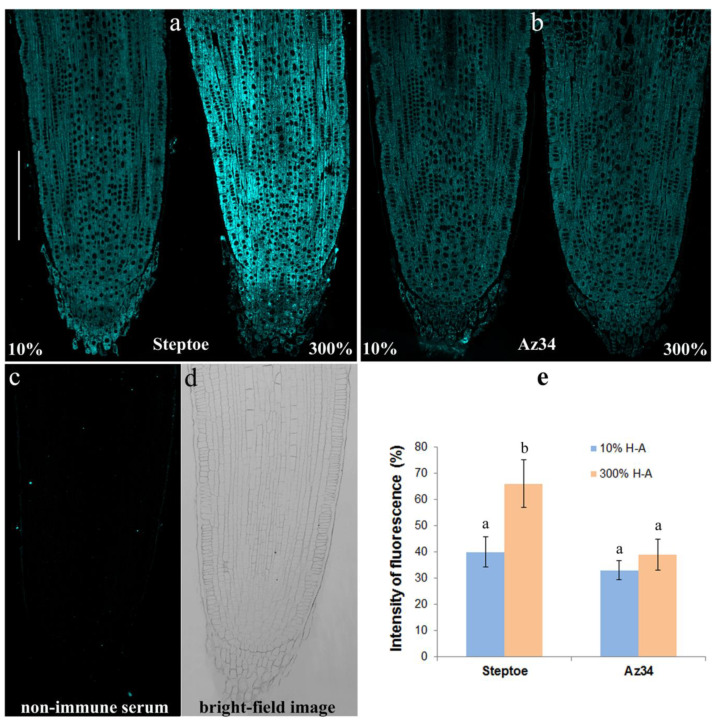
Cytokinin localization in the root tips of Steptoe (**a**) and Az34 (**b**) 6-day-old plants grown on either 300% or 10% of H-A solution. Immunohistochemical control with non-immune serum (**c**) and the bright-light image of the root tips (**d**). Fluorescence for cytokinins (means ± SE, arbitrary units, maximal fluorescence taken as 100%, minimal as 0%) (**e**). Statistically different means (n = 9) are indicated by different letters (*p* < 0.05, ANOVA, Duncan’s test). Scale bar is 200 µM.

The immunohistochemical study of sections of the roots showed increased fluorescence brightness in the case of Steptoe plants grown at supra-optimal compared to low concentration of mineral nutrients (Figure 7a), while no difference in root fluorescence between plants supplied with either level of mineral nutrients were detected in the case of Az34 (Figure 7b). The specificity of the method was confirmed by the absence of fluorescence in control sections processed with non-immune serum (Figure 7c,d). Statistical significance of the difference between section in fluorescence was confirmed by the results of the estimation average fluorescence intensity of the images taken with the confocal microscope with the ImageJ software (Figure 7e).

## 4. Discussion

Root shortening of Steptoe plants grown on supra-optimal (300%) H-A solution was accompanied by and was likely to be due to accumulation of cytokinins detected, both in whole roots (Figure 1, Figure 2 and Figure 3) and their tips (Figure 7a). These results are in accordance with the known inhibitory effect of cytokinins on root growth [26]. The relationship between these effects is confirmed by the data showing that 300% H-A does not affect the root length of Az34 plants, which is apparently associated with the absence of the accumulation of cytokinins in the roots of mutant plants. 

Although, in contrast to the response of Steptoe, high nutrient concentration did not increase, but decreased concentration of zeatin and its nucleotide in the roots of Az34, the changes in concentration of these hormones were not accompanied by any changes in the root length. The absence of root elongation response correlated with the absence of the changes in zeatin riboside concentration in the roots of Az34. These results suggest that zeatin riboside may play a more important role than was previously suggested, which is in accordance with the recent review, showing that ribosides of cytokinins are not merely the transport form of these hormones but are active in regulating the development and environmental responses of plants [27]. 

In the present work, we analyzed cytokinins using antibodies raised against zeatin riboside, having high affinity to zeatin derivatives and low cross-reactivity with derivatives of isopentenyladenine (iPA). This choice was dictated by the previous data, showing accumulation of zeatin and its riboside in roots of wheat treated with iP, explained by hydroxylation of the iP and its conversion to zeatin [28]. Still, further experiments should check the possible involvement of iP and its ribosides in the control of barley root growth.

The increased staining for cytokinins found in the nutrient-rich Steptoe root tips is very important in explaining the slowed root growth as the root growth process takes place in this part of the root. The immunohistochemical technique showed higher cytokinin content both in dividing and elongating root cells, which is in accordance with the ability of cytokinins to inhibit both division [29] and elongation of root cells [30], resulting in the inhibition of their growth. Similar results have been obtained previously in experiments with split roots divided between compartments with different concentrations of ions, which showed accumulation of cytokinins in the roots contacting with concentrated solution accompanied by inhibition of their growth [14]. However, the mechanism of cytokinin accumulation in those experiments remained unclear. Present experiments show that this may be due to inhibition of cytokinin export from the roots. This is suggested by the coincidence of cytokinin accumulation in the roots of nutrient-rich Steptoe plants, with the decline in cytokinin level in their shoots. Furthermore, reduced cytokinin concentration found in the xylem sap of these plants may serve as direct evidence of a decrease in the transport of cytokinins from the roots. Cytokinins delivery from roots to shoots of nutrient-rich barley plants was decreased to an even greater extent than their concentration due to a lower transpiration rate (Table 1). Decreased transpiration of these plants can be explained by the lowered concentration of cytokinins found in their shoots since these hormones are known to maintain transpiration [31].

Decreased delivery of hormones from roots to shoots is mainly explained by inhibition of their synthesis in the roots [7]. However, this explanation cannot be applied in our case, since accumulation of cytokinins found in the roots of nutrient-rich Steptoe plants argues against it. The discovery of cytokinin transporters ([32] and references therein) makes it possible to explain the mechanism of direct regulation of cytokinin delivery from roots to shoots. It has been shown previously that inhibition secondary active transmembrane transport by application of protonophore CCCP results in a decline in cytokinin concentration in the roots, suggesting that its active uptake by roots prevented cytokinin export from roots to shoots [22]. Cytokinins up taken from apoplast are likely to originate either from root cells capable to synthesize them [11] or delivered from shoots through phloem [27,28]. Present research does not allow us to identify exact cytokinin transporters involved in the control of cytokinin accumulation in the roots under supra-optimal concentration of mineral nutrients. Nevertheless, proton gradients across membranes are known to be important for secondary active trans-membrane transport. In present experiments, addition of protonophore CCCP prevented cytokinin accumulation in the roots of nutrient-rich Steptoe plants, suggesting involvement of a secondary active process of cytokinin uptake by root cells in the accumulation of this hormone by barley plants grown in the presence of supra-optimal concentration of mineral nutrients. Retention of cytokinins in root cells of Steptoe plants due to activity of transporters for cytokinins enabling their energized uptake by cells against a concentration gradient prevents their outflow to the shoots with transpiration stream. 

It is important to note that neither accumulation of cytokinins in the roots nor inhibition of their elongation was detected in ABA-deficient barley mutant (Figure 1, Figure 2 and Figure 3). Low ABA concentration in these plants is due to mutation of the gene-encoding molybdenum cofactor of aldehyde oxidase catalyzing ABA synthesis from its aldehyde [11]. ABA concentration was lower in Az34 than in Steptoe plants, and, unlike their parental cultivar, concentration of ABA did not increase in the roots of nutrient-rich barley plants (Figure 6). These results suggest the importance of plant capacity to accumulate ABA for their root cytokinin and growth responses. High concentrations of nitrates have been shown to increase ABA concentration in the roots of maize plants, which was shown to be due to the release of free ABA from its conjugates [33]. It is not excluded that this mechanism was operating in the present experiments. However, the absence of significant ABA accumulation in the Az34 plants with decreased activity of ABA aldehyde oxidase [34] suggests implication of ABA synthesis in the accumulation of this hormone in response to supra-optimal concentrations of mineral nutrients. Withdrawal of phosphates from the nutrient solution has been shown to result in a decline in cytokinin concentration in the root tips due to the ABA-induced down-regulation of *HvIPT-*1 gene involved in the control of cytokinin synthesis in the roots of barley plants [11]. This mechanism is not likely to operate in the roots of nutrient-rich barley plants, since concentration of cytokinins was increased and not decreased by supra-optimal concentration of mineral nutrients. Nitrates are known to up-regulate *IPT* genes both in Arabidopsis and maize [7], and it is obvious that this effect overcame possible ABA-induced down-regulation of the genes in the nutrient-rich Steptoe plants, resulting in the high concentration of cytokinins in the roots of the plants. Increased concentration of ABA accompanied by decreased cytokinin concentration was detected in the shoots of plants exposed either to the deficit of nitrates or their supra-optimal concentration [35], and these effects are not likely to reflect mere coincidence but ABA action on cytokinin synthesis and transport from roots to shoots. ABA is likely to act in a different way in the nutrient-rich plants compared to nutrient-starved plants, inhibiting cytokinin transport from roots to shoots, thereby resulting in cytokinin accumulation in the roots and inhibition of their elongation. 

## 5. Conclusions

**Figure 8:**
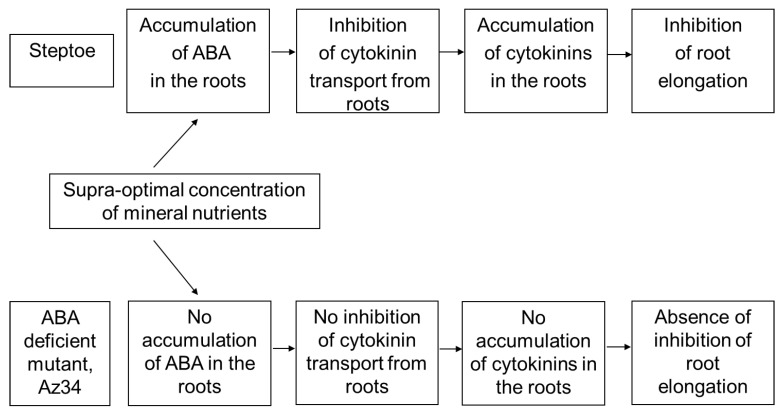
Responses of ABA-deficient barley mutant (Az34) and its parental genotype Steptoe to supra-optimal concentration of mineral nutrients.

Comparison of the effects of supra-optimal concentration of mineral nutrients on ABA-deficient barley mutant and its parental cultivar showed dependence of both root growth and cytokinin responses on the capacity of plants to produce ABA. In Steptoe plants capable of accumulating ABA, supra-optimal concentration of mineral nutrients resulted in a decline in cytokinin export from roots to shoots, bulk accumulation of cytokinins in the roots, and their increased abundance in the growing tips of roots, bringing about inhibition of root elongation. All those effects were absent in ABA-deficient barley plants. The results are presented as a scheme in Figure 8.

## Figures and Tables

**Table 1:** Transpiration and cytokinin (CK) delivery from roots to shoot of 6-day-old barley wild type (St) and its ABA-deficient mutant (Az34) growing at 10% of supra-optimal (300%) Hoagland-Arnon solution (H-A).

Genotype and Treatment	Transpiration, mg H_2_O h^−1^ plant^−1^	Delivery, ng CK h^−1^ plant^−1^		
		Z	ZR	ZN
St, 10% H-A	51.6 ± 2.8 ^a^	0.20 ± 0.01 ^b^	0.20 ± 0.01 ^b^	0.12 ± 0.02 ^b^
St, 300% H-A	47.7 ± 1.9 ^a^	0.17 ± 0.02 ^a^	0.11 ± 0.01 ^a^	0.06 ± 0.01 ^a^
Az34, 10% H-A	61.5 ± 2.7 ^b^	0.29 ± 0.02 ^c^	0.22 ± 0.03 ^b^	0.18 ± 0.01 ^c^
Az34, 300% H-A	60.5 ± 2.5 ^b^	0.25 ± 0.01 ^bc^	0.20 ± 0.2 ^b^	0.15 ± 0.01 ^bc^

Statistically different means (n = 9) are indicated by different letters (*p* < 0.05, ANOVA, Duncan’s test).

## Data Availability

The data presented in this study are available in the graphs and tables provided in the manuscript.

## References

[1] Li X., Zeng R., Liao H. (2016). Improving crop nutrient efficiency through root architecture modifications. J. Integr. Plant Biol..

[2] Lynch J.P. (2019). Root Phenotypes for improved nutrient capture: An underexploited opportunity for global agriculture. New Phytol..

[3] Forde B.G. (2002). Local and long-range signaling pathways regulating plant responses to nitrate. Annu. Rev. Plant Biol..

[4] Ruffel S., Krouk G., Ristova D., Shasha D., Birnbaum K.D., Coruzzi G.M. (2011). Nitrogen economics of root foraging: Transitive closure of the nitrate–cytokinin relay and distinct systemic signaling for N supply vs. demand. Proc. Natl. Acad. Sci. USA.

[5] Hirose N., Takei K., Kuroha T., Kamada-Nobusada T., Hayashi H., Sakakibara H. (2008). Regulation of cytokinin biosynthesis, compartmentalization, and translocation. J. Exp. Bot..

[6] Takei K., Sakakibara H., Sugiyama T. (2001). Identification of genes encoding adenylate isopentenyltransferase, a cytokinin biosynthesis enzyme, in Arabidopsis thaliana. J. Biol. Chem..

[7] Takei K., Ueda N., Aoki K., Kuromori T., Hirayama T., Shinozaki K., Yamaya T., Sakakibara H. (2004). AtIPT3 is a key determinant of nitrate-dependent cytokinin biosynthesis in Arabidopsis. Plant Cell Physiol..

[8] Gojon A., Krouk G., Perrine-Walker F., Laugier E. (2011). Nitrate transceptor(s) in plants. J. Exp. Bot..

[9] Wang R., Xing X., Wang Y., Tran A., Crawford N.M. (2009). A genetic screen for nitrate regulatory mutants captures the nitrate transporter gene NRT1.1. Plant Physiol..

[10] Korobova A.V., Akhiyarova G.R., Fedyaev V.V., Farkhutdinov R.G., Veselov S.Y., Kudoyarova G.R. (2019). Participation of nitrate sensor NRT1.1 in the control of cytokinin level and root elongation under normal conditions and nitrogen deficit. Mosc. Univ. Biol. Sci. Bull..

[11] Vysotskaya L., Akhiyarova G., Feoktistova A., Akhtyamova Z., Korobova A., Ivanov I., Dodd I., Kuluev B., Kudoyarova G. (2020). Effects of Phosphate Shortage on Root Growth and Hormone Content of Barley Depend on Capacity of the Roots to Accumulate ABA. Plants.

[12] Vysotskaya L.B., Trekozova A.W., Kudoyarova G.R. (2016). Effect of phosphorus starvation on hormone content and growth of barley plants. Acta Physiol. Plant..

[13] Kudoyarova G.R., Dodd I.C., Veselov D.S., Rothwell S.A., Veselov S.Y. (2015). Common and specific responses to availability of mineral nutrients and water. J. Exp. Bot..

[14] Korobova A.V., Ivanov I.I., Akhiyarova G.R., Veselov D.S., Kudoyarova G.R., Veselov S.Y. (2019). Influence of macroelements’ uneven distribution on the content of hormones and extension of the roots in wheat plants. Russ. J. Plant Physiol..

[15] Werner T., Motyka V., Laucou V., Smets R., Van Onckelen H., Schmulling T. (2003). Cytokinin-deficient transgenic Arabidopsis plants show multiple developmental alterations indicating opposite functions of cytokinins in the regulation of shoot and root meristem activity. Plant Cell.

[16] Vysotskaya L.B., Korobova A.V., Veselov S.Y., Dodd I.C., Kudoyarova G.R. (2009). ABA mediation of shoot cytokinin oxidase activity: Assessing its impacts on cytokinin status and biomass allocation of nutrient deprived durum wheat. Funct. Plant Biol..

[17] Harris J.M., Ondzighi-Assoume C.A. (2017). Environmental nitrate signals through abscisic acid in the root tip. Plant Signal. Behav..

[18] Li X., Chen L., Forde B.G., Davies W.J. (2017). The biphasic root growth response to abscisic acid in Arabidopsis involves interaction with ethylene and auxin signalling pathways. Front. Plant Sci..

[19] Spollen W.G., Lenoble M.E., Samuels T.D., Bernstein N., Sharp R.E. (2000). Abscisic acid accumulation maintains maize primary root elongation at low water potentials by restricting ethylene production. Plant Physiol..

[20] Vysotskaya L.B., Korobova A.V., Kudoyarova G.R. (2008). Abscisic acid accumulation in the roots of nutrient-limited plants: Its impact on the differential growth of roots and shoots. J. Plant Physiol..

[21] de Groot C.C., Marcelis L.F.M., van den Boogaard R., Kaiser W.M., Lambers H. (2003). Interaction of nitrogen and phosphorus nutrition in determining growth. Plant Soil.

[22] Kudoyarova G.R., Korobova A.V., Akhiyarova G.R., Arkhipova T.N., Zaytsev D.Y., Prinsen E., Egutkin N.L., Medvedev S.S., Veselov S.Y. (2014). Accumulation of cytokinins in roots and their export to the shoots of durum wheat plants treated with the protonophore carbonyl cyanide m-chlorophenylhydrazone (CCCP). J. Exp. Bot..

[23] Arkhipova T.N., Melentiev A.I., Martynenko E.V., Kudoyarova G.R., Veselov S.U. (2005). Ability of bacterium *Bacillus subtilis* to produce cytokinins and to influence the growth and endogenous hormone content of lettuce plants. Plant Soil.

[24] Veselov S.U., Kudoyarova G.R., Egutkin N.L., Gyuli-Zade V.G., Mustafina A.R., Kof E.K. (1992). Modified solvent partitioning scheme providing increased specificity and rapidity of immunoassay for indole 3-acetic acid. Physiol. Plant..

[25] Vysotskaya L.B., Akhiyarova G.R., Sharipova G.V., Dedova M.A., Veselov S.Y., Zaitsev D.Y., Kudoyarova G.R. (2015). The influence of local IPT gene induction in roots on content of cytokinins in cells of tobacco leaves. Cell Tissue Biol..

[26] Werner T., Nehnevajova E., Köllmer I., Novák O., Strnad M., Krämer U., Schmülling T. (2010). Root-specific reduction of cytokinin causes enhanced root growth, drought tolerance, and leaf mineral enrichment in Arabidopsis and tobacco. Plant Cell.

[27] Nguyen H.N., Nguyen T.Q., Kisiala A.B., Neil Emery R.J. (2021). Beyond transport: Cytokinin ribosides are translocated and active in regulating the development and environmental responses of plants. Planta.

[28] Veselov S.Y., Timergalina L.N., Akhiyarova G.R., Kudoyarova G.R., Korobova A.V., Ivanov I.I., Arkhipova T.N., Prinsen E. (2018). Study of cytokinin transport from shoots to roots of wheat plants is informed by a novel method of differential localization of free cytokinins bases or their ribosylated forms by means of their specific fixation. Protoplasma.

[29] Ivanov V.B., Filin A.N. (2017). Cytokinins regulate root growth through its action on meristematic cell proliferation but not on the transition to differentiation. Funct. Plant Biol..

[30] Růžička K., Šimášková M., Duclercq J., Petrášek J., Zažímalová E., Simon S., Friml J., Van Montagu M.C.E., Benková E. (2009). Cytokinin regulates root meristem activity via modulation of the polar auxin transport. Proc. Natl. Acad. Sci. USA.

[31] Farber M., Attia Z., Weiss D. (2016). Cytokinin activity increases stomatal density and transpiration rate in tomato. J. Exp. Bot..

[32] Duran-Medina I., Diaz-Ramirez D., Marsch-Martinez N. (2017). Cytokinins on the Move. Front. Plant Sci..

[33] Ondzighi-Assoume C.A., Chakraborty S., Harris J.M. (2016). Environmental nitrate stimulates abscisic acid accumulation in Arabidopsis root tips by releasing it from inactive stores. Plant Cell.

[34] Walker-Simmons M., Kudrna D.A., Warner R.L. (1989). Reduced accumulation of ABA during water stress in a molybdenum cofactor mutant of barley. Plant Physiol..

[35] Wilkinson S., Hartung W. (2009). Food production: Reducing water consumption by manipulating long-distance chemical signalling in plants. J. Exp. Bot..

